# Neoadjuvant chemotherapy in locally advanced nasopharyngeal carcinoma: Defining high-risk patients who may benefit before concurrent chemotherapy combined with intensity-modulated radiotherapy

**DOI:** 10.1038/srep16664

**Published:** 2015-11-13

**Authors:** Xiao-Jing Du, Ling-Long Tang, Lei Chen, Yan-Ping Mao, Rui Guo, Xu Liu, Ying Sun, Mu-Sheng Zeng, Tie-Bang Kang, Jian-Yong Shao, Ai-Hua Lin, Jun Ma

**Affiliations:** 1 Department of Radiation Oncology, Sun Yat-sen University Cancer Center; State Key Laboratory of Oncology in South China; Collaborative Innovation Center for Cancer Medicine, No. 651 Dongfeng Road East, Guangzhou 510060, China; 2 Sun Yat-sen University Cancer Center; State Key Laboratory of Oncology in South China; Collaborative Innovation Center for Cancer Medicine, No. 651 Dongfeng Road East, Guangzhou 510060, China; 3 Department of Molecular Diagnostics, Sun Yat-sen University Cancer Center; State Key Laboratory of Oncology in South China; Collaborative Innovation Center for Cancer Medicine, No. 651 Dongfeng Road East, Guangzhou 510060, China; 4 Department of Medical Statistics and Epidemiology, School of Public Health, Sun Yat-sen University, No. 74 Zhongshan Road, Guangzhou 510060, China

## Abstract

The purpose of this study was to create a prognostic model for distant metastasis in patients with locally advanced NPC who accept concurrent chemotherapy combined with intensity-modulated radiotherapy (CCRT) to identify high-risk patients who may benefit from neoadjuvant chemotherapy (NACT). A total of 881 patients with newly-diagnosed, non-disseminated, biopsy-proven locoregionally advanced NPC were retrospectively reviewed; 411 (46.7%) accepted CCRT and 470 (53.3%) accepted NACT followed by CCRT. Multivariate analysis demonstrated N2–3 disease, plasma Epstein–Barr virus (EBV) DNA > 4000 copies/mL, serum albumin ≤46 g/L and platelet count >300 k/cc were independent prognostic factors for distant metastasis in the CCRT group. Using these four factors, a prognostic model was developed, as follows: 1) low-risk group: 0–1 risk factors; and 2) high-risk group: 2–4 risk factors. In the high-risk group, patients who accepted NACT + CCRT had significantly higher distant metastasis-free survival and progression-free survival rates than the CCRT group (*P* = 0.001; *P* = 0.011). This simple prognostic model for distant metastasis in locoregionally advanced NPC may facilitate with the selection of high-risk patients who may benefit from NACT prior to CCRT.

Nasopharyngeal carcinoma (NPC) is endemic in Southeast Asia, North Africa, Alaska and the Mediterranean basin^1^. Due to its silent, deep-seated location and mild, non-specific symptoms, early detection is a challenge; 60–70% patients present with locally advanced NPC at diagnosis^2^. The standard therapy for non-disseminated NPC is radiotherapy; however, this strategy successfully controls disease in only 67%–77% of patients with advanced disease^3^. Intensity-modulated radiation therapy (IMRT) is now the primary radiotherapy modality in NPC as it provides better dose distribution and locoregional control^4^,^5^. Additionally, several clinical trials and meta-analysis demonstrated chemotherapy administered concurrently with radiotherapy (CCRT) is the most effective treatment and improves overall survival^6^,^7^,^8^,^9^,^10^. Nevertheless, over 20% of patients still experience distant metastasis after CCRT, necessitating exploration of other intensive treatment modalities for NPC^11^.

Addition of neoadjuvant chemotherapy (NACT) before CCRT may be a reasonable approach. Theoretically, NACT could reduce the tumor burden and kill occult micro-metastases, which may improve survival. A recent meta-analysis revealed NACT significantly reduced the risk of distant metastasis in NPC^12^. But published single arm or randomized phased II studies regarding the efficacy of NACT followed by CCRT in locally advanced NPC have provided conflicting results^13^,^14^,^15^. One possible reason for the lack of benefit is due to the inclusion criteria used in those studies, which was mainly based on patients’ clinical stages. However, the present NPC staging system is restricted in its diagnostic reach to the anatomical extent of the tumors, and may not accurately categorize patients at high risk of distant metastasis. As NACT may induce an unnecessary financial burden and delay CCRT, it is of utmost importance to identify high-risk patients who may obtain benefit from NACT before treatment.

Although the Tumor, Node, Metastasis (TNM) staging system is widely used to predict prognosis and guide therapy, accumulating data suggests circulating Epstein–Barr virus (EBV) DNA and several other serum markers as prognostic factors for distant metastasis in NPC^16^,^17^,^18^,^19^,^20^. Therefore, we retrospectively analyzed a large cohort of patients to evaluate the prognostic value of pretreatment clinical and laboratory factors and construct a prognostic score model to facilitate pretreatment decision-making regarding NACT in NPC.

## Methods and Materials

### Patients

We reviewed all cases of newly-diagnosed, biopsy-proven, non-metastatic NPC treated at Sun Yat-sen University Cancer Center using IMRT between October 2009 and February 2012. In all, 1811 cases were evaluated, of whom 1330 (73.4%) were diagnosed with stage III-IVb disease according to the 7th edition of the International Union against Cancer/American Joint Committee on Cancer (UICC/AJCC) staging system^21^. Of these, 1044/1330 (78.5%) were treated with CCRT or NACT + CCRT; 163/1044 (15.6%) cases were subsequently eliminated due to incomplete laboratory data. Therefore, 881 patients were included in this analysis. The study was approved by the Institutional Review Board of Sun Yat-sen University Cancer Center, and was conducted in accordance with the Good Clinical Practice guideline. Informed consent was obtained from each patient for their consent to have their information used in research without affecting their treatment option or violating their privacy.

Two experienced radiologists separately evaluated all MRI scans to minimize heterogeneity, and two physicians specializing in head and neck cancer restaged all patients according to 7th edition of the UICC/AJCC. Any disagreements were resolved by consensus.

### Laboratory measurements

Plasma EBV DNA, serum lactate dehydrogenase (LDH), serum alkaline phosphatase (ALP), serum albumin, leukocytes, hemoglobin and platelets were measured in all patients at the same time within 2 weeks before therapy. Blood counts were performed using a Sysmex XE-5000 automated hematology analyzer (Sysmex, Kobe, Japan). Serum LDH, ALP and albumin were measured using an automated immunoturbidmetric analyzer (7600-020; Hitachi High-Technologies, Tokyo, Japan). Patient plasma EBV DNA was measured by real-time quantitative PCR targeting the EBV BamH I-W region^22^,^23^.

### Treatment

All patients were treated using IMRT with one fraction daily 5 days per week. Target volumes were delineated according to International Commission on Radiation Units and Measurements Reports 50 and 62. Clinical target volumes (CTV) were individually delineated based on the tumor invasion pattern. The prescribed radiation dose was: a total of 68–70 Gy in 30–33 fractions at 2.13–2.27 Gy/fraction to the planning target volume (PTV) of the gross tumor volume of the primary (GTV-P), 60–68 Gy to the nodal gross tumor volume PTV (GTV-N), 60 Gy to the PTV of CTV-1 (high-risk regions), and 54–56 Gy to the PTV of CTV-2 (low-risk regions and neck nodal regions). In total, 411/881 (46.7%) patients received CCRT, and 470 (53.3%) received NACT + CCRT. NACT was a platinum-based regimen of two or three drugs every 3 weeks for two or three cycles; 796/881 (90.4%) patients received a single-drug platinum-based CCRT regimen every 3 weeks for at least 2 cycles, or weekly for at least 3 cycles.

### Follow-up

After treatment, patients were examined every 3 months during the first 2 years, and every 6 months thereafter for up to 5 years or until death. Median follow-up was 38.7 (range, 1.3–60.2) months. No patients were lost to follow-up. The following end points (time to first defining event) were assessed: distant metastasis-free survival (DMFS), progression-free survival (PFS), overall survival (OS) and local relapse-free survival (LRFS).

### Statistical analysis

All calculations were performed using Statistical Package for the Social Sciences, version 20.0 (SPSS, Chicago, IL, USA). Grouping by EBV DNA, leukocyte count, platelet count, hemoglobin, LDH and ALP was performed using standard or published thresholds^16^,^17^,^18^,^19^,^20^. Serum albumin was analyzed as a binary variable using the median value of the CCRT group as a cut-off (≥median of high-serum albumin group and < median of low serum albumin group). The Chi-square test was used to analyze differences between the CCRT and NACT + CCRT groups. Two-tailed *P*-values < 0.05 were considered significant.

*Step 1: Survival prediction.* The CCRT group was used to determine the prognostic significance of pretreatment clinical and laboratory factors for distant metastasis in univariate (Kaplan–Meier method and log-rank test) and multivariable analysis (Cox proportional hazards model to test independent significance by backward elimination of insignificant explanatory variables).

*Step 2: Model construction.* A prognostic score model was created based on the independent prognostic factors identified in the CCRT group. The maximum score for each patient was equal to the total number of risk factors. The cut-off score to define the high-risk and low-risk groups for DMFS was identified using receiver-operating characteristic (ROC) curve analysis.

*Step 3: Stratification survival analysis.* The efficacy of NACT was assessed for each stratification of the entire cohort dichotomized by each individual prognostic factor and the prognostic score model.

*Step 4: Multivariate survival analysis in the high-risk group.* Multivariate analysis of the high-risk group was performed to confirm the benefit of NACT in addition to CCRT while controlling for host, tumor and laboratory parameters.

## Results

### Clinicopathological features and treatment outcomes

The clinicopathological characteristics of the 881 patients are presented in Table 1. Median age at diagnosis was 45 (range, 14–77) years; 98.9% of patients had type II or III disease, based on the World Health Organization (WHO) criteria.

Patients with stage IV, T4 or N2–3 disease were more likely to receive NACT than patients with stage III (*P* < 0.001), T1–3 (*P* < 0.001) or N0–1 (*P* = 0.002) disease. High pretreatment plasma EBV DNA (> 4,000 copies/ml; *P* < 0.001), leukocyte count (*P* = 0.015), platelet count (*P* = 0.012) and serum LDH (*P* = 0.001), and low pretreatment albumin (*P* = 0.001) were significantly associated with NACT.

The biases towards selecting patients with bulky tumors or high EBV DNA for more aggressive treatment reflects the clinical decision-making preferences during the study period. Despite these variations, there was no significant difference in any end-point between treatment groups (Table 1).

### Prognostic factors for metastasis in the CCRT group

Univariate analysis identified N2–3 disease (*P* = 0.003), plasma EBV DNA (*P* = 0.003), serum albumin (*P* = 0.009), leukocyte count (*P* = 0.012) and platelet count (*P* = 0.016) as significant prognostic factors for DMFS. Multivariate analysis confirmed all of these factors, except leukocyte count, were independent prognostic factors for DMFS in locally advanced NPC after CCRT (Table 2).

### Prognostic score model for the CCRT group

**Figure 1:**
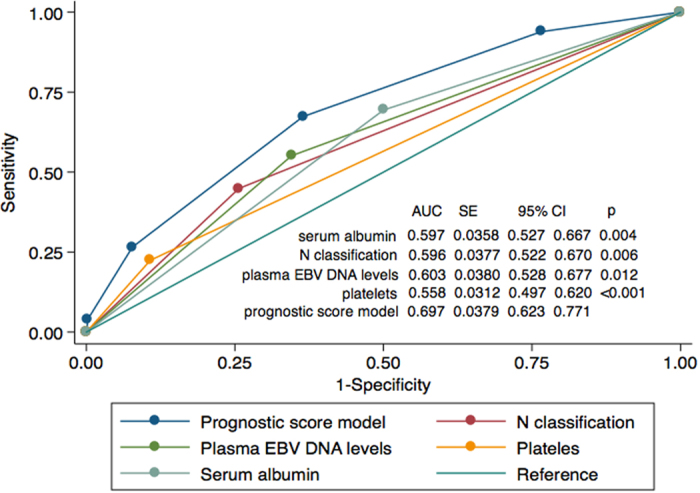
Receiver-operating characteristic curves for distant metastasis in locally advanced NPC after concurrent chemotherapy (*n* = 411) based on the individual prognostic factors and prognostic score model. *P*-values vs. prognostic score model.

We constructed a prognostic score model for DMFS in patients with locally advanced NPC who accepted CCRT. Patients were sub-grouped by N classification, pretreatment plasma EBV DNA, serum albumin and platelet count. If a risk factor was present, a score of 1 was recorded (maximum score, 4). The ROC curves are shown in Fig. 1. The area under the curve (AUC) for the prognostic score model was 0.697; a score of 1.5 resulted in a sensitivity of 0.67 and specificity of 0.64 for DMFS.

Thus, two risk stratification groups were obtained: 1) low-risk: total score 0–1 (246 patients); and 2) high-risk: total score 2–4 (165 patients). The 3-year DMFS rates for the low and high-risk groups were 93.1%, and 80.3% (*P* < 0.001) and the 3-year PFS rates were 88.5% and 72.5% (*P* < 0.001), respectively.

### Stratification survival analysis

**Figure 2:**
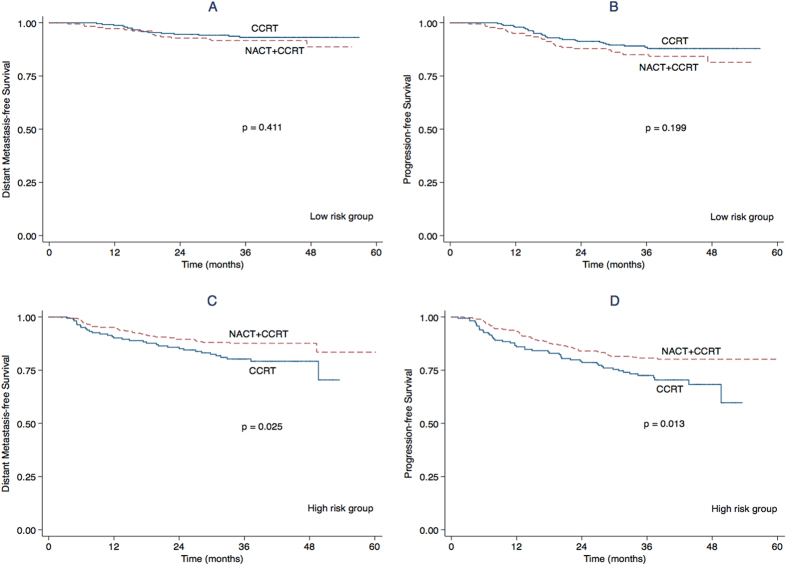
Kaplan-Meier distant metastasis-free survival curves and progression-free survival curves for patients with locally advanced NPC in the low-risk (A,B), and high-risk groups (C,D) stratified by the CCRT and NACT + CCRT.

Based on the prognostic score model, we stratified all 881 patients with locally advanced NPC as low-risk and high-risk. High-risk patients who accepted NACT + CCRT had significantly better DMFS and PFS than high-risk patients who accepted CCRT (*P* = 0.025; *P* = 0.013; Fig. 2). However, NACT provided no additional survival advantage in the low-risk group or when patients were stratified by the individual prognostic factors (Table 3).

### Multivariate analysis of the high-risk group

The covariates listed in Table 2 were included in multivariate analysis of the 454 patients in the high-risk group. Patients in the high-risk group who accepted NACT + CCRT had significantly higher DMFS and PFS (*P* = 0.001; *P* = 0.011) than high-risk patients who accepted CCRT alone (Table 4).

## Discussion

Ongoing phase III trials (e.g. NCT01245959, NCT00828386, NCT01536223, NCT00201396) are examining the most effective NACT regimens to improve the relatively poor prognosis of locoregionally advanced NPC. To our knowledge, this is the first attempt to design a prognostic score model to select high-risk patients with locoregionally advanced NPC who may benefit from NACT before CCRT.

Some results of this study are consistent with previous findings and consensus. Bulky or extensive nodal disease were associated with a poorer DMFS after CCRT, indicating the need for more intensive combined primary treatments. However, in contrast to a previous study^2^, T classification was not prognostic for DMFS, which is reasonable given CCRT was used in this study. As concurrent chemotherapy with IMRT can achieve excellent locoregional control and OS in NPC^4^,^5^,^6^,^7^,^8^,^9^,^10^,^11^, the prognostic effect of T classification may have become less relevant; T classification is generally considered an indicator of local invasion.

High pretreatment plasma EBV DNA was validated as a prognostic factor for DMFS after CCRT in locoregionally advanced NPC. EBV and its gene products play a pathogenic role and have prognostic value in the non-keratinizing subtypes of NPC in patients from the endemic region. Numerous groups have confirmed circulating EBV DNA correlates with tumor stage, presence of residual disease or metastasis, and OS in NPC^22^,^24^,^25^,^26^. Additionally, plasma EBV DNA has prognostic value for poorer OS in stage III and IV NPC, indicating the potential of this biomarker to complement the TNM classification during treatment planning^24^.

Furthermore, a high pretreatment platelet count and lower serum albumin were associated with unfavorable DMFS after CCRT. These host-related factors reflect host-tumor interactions. Platelets are involved in hemostasis, angiogenesis, inflammation and wound healing, and may play a role in cancer biology by promoting primary tumor growth via facilitating angiogenesis and tumor invasion via platelet-derived microparticles or thrombin activity^27^. Platelets may also surround and protect circulating tumor cells from elimination by natural killer cells, and thus promote distant metastasis^28^. A high platelet count is an unfavorable prognostic factor in several solid tumors, including NPC^20^,^27^,^28^. Several studies have investigated anti-platelet therapy in cancer; nonsteroidal antiinflammatory drugs (NSAIDs) and selective inhibitors of arachidonic acid cyclooxygenase-2 (COXIBs) have been reported to effectively inhibit cancer initiation and progression^29^.

Serum albumin is regularly used as a biomarker of long-term nutritional status, and is also known to correlate with systemic inflammation, stabilize cell growth and DNA replication, buffer a variety of biochemical changes, and prevent development of sex hormone-induced cancers^30^. Associations have been reported between low serum albumin and increased disease severity, higher risk of disease progression and poorer OS in cancer^30^. Additionally, although most patients have normal serum albumin values at diagnosis, a lower pretreatment serum albumin to globulin level (<1.4) was associated with poorer OS in NPC^31^.

The major challenges in NPC are assessment of the risk of metastasis and development of preventive treatments. It has been shown inadequate to apply only the TNM staging system for treatment guidance, and use of biomarkers would probably enhance the power of clinical trials to obtain positive results. For example, the phase III trials (NCT00370890) is designed to evaluate the benefit of adjuvant chemotherapy in high-risk NPC patients, identified with detectable plasma EBV DNA six weeks after chemo-radiotherapy. However, up until now, no similar-designed study has been conducted regarding the use of NACT yet. Our prognostic score model combines several pretreatment clinical variables with the clinical implications of both tumor burden and host response. Using this model, patients could be separated into low-risk and high-risk groups with different survival outcomes and responses to NACT. Therefore, the prognostic model may complement the current clinical staging system and enable identification of patients who may benefit from more intensive therapy in addition to CCRT.

The major limitation of this study is its retrospective single-center design. However, we endeavored to control biases by striving to review all patients treated with IMRT during the study period. Nevertheless, a prospective study is necessary to validate the prognostic model.

In conclusion, the prognostic score model based on N classification, pretreatment plasma EBV DNA, platelet count and serum albumin provides a useful method of selecting patients with locoregionally advanced NPC who may benefit from more intensive treatment. Furthermore, addition of NACT to the standard CCRT regimen may provide most benefit in patients with two or more risk factors.

## Additional Information

**How to cite this article**: Du, X.-J. *et al.* Neoadjuvant chemotherapy in locally advanced nasopharyngeal carcinoma: Defining high-risk patients who may benefit before concurrent chemotherapy combined with intensity-modulated radiotherapy. *Sci. Rep.* **5**, 16664; doi: 10.1038/srep16664 (2015).

## Figures and Tables

**Table 1:** Characteristics of the 881 patients with NPC.

Characteristic	CCRT	NACT + CCRT	*P*-value
	No. of patients (%)	No. of patients (%)	
**Total**	411	470	
**Age, years**			0.083
≤50	278 (67.6)	343 (73.0)	
>50	133 (32.4)	127 (27.0)	
**Sex**			0.699
Male	312 (75.9)	362 (77.0)	
Female	99 (24.1)	108 (23.0)	
**T classification**			<0.001
T1–3	338 (82.2)	315 (67.0)	
T4	73 (17.8)	155 (33.0)	
**N classification**			0.002
N0–1	296 (72.0)	292 (62.1)	
N2–3	115 (28.0)	178 (37.9)	
**Clinical stage**			<0.001
III	314 (76.4)	252 (53.6)	
IV	97 (23.6)	218 (46.4)	
**Serum EBV DNA, copies/mL**			<0.001
≤4000	259 (63.0)	186 (39.6)	
>4000	152 (37.0)	284 (60.4)	
**Leukocytes, k/cc**			0.015
≤10	384 (93.4)	417 (88.7)	
>10	27 (6.6)	53 (11.3)	
**Hemoglobin, g/L**			0.513
≤120	26 (6.3)	35 (7.4)	
>120	385 (93.7)	435 (92.6)	
**Platelets, k/cc**			0.012
≤300	361 (87.8)	384 (81.7)	
>300	50 (12.2)	86 (18.3)	
**Serum albumin, g/L**			0.001
≤ 46	215 (52.3)	300 (63.8)	
> 46	196 (47.7)	170 (36.2)	
**Serum alkaline phosphatase, U/L**			0.254
≤110	389 (94.6)	436 (92.8)	
>110	22 (5.4)	34 (7.2)	
**Serum lactate dehydrogenase, U/L**			0.001
≤245	399 (97.1)	431 (91.7)	
>245	12 (2.9)	39 (8.3)	
**Progression-free survival**			0.784
Events	76 (18.5)	85 (18.1)	
Rate at 3 years, %	82.0	82.3	
**Distant metastasis-free survival**			0.627
Events	49 (11.9)	52 (11.1)	
Rate at 3 years, %	88.0	89.2	
**Local relapse-free survival**			0.852
Events	31 (7.5)	38 (8.1)	
Rate at 3 years, %	93.1	91.9	
**Overall survival**			0.801
Events	32 (7.8)	40 (8.5)	
Rate at 3 years, %	93.9	92.4	

Abbreviations: CCRT, concurrent chemoradiotherapy; NACT, neoadjuvant chemotherapy.

**Table 2:** Survival analysis of risk factors for distant metastasis in patients with NPC who accepted CCRT (*n* = 411).

Factor	Distant metastasis-free survival rate at 3 years (%)	Univariate analysis	Multivariate analysis	*P*-value
		*P* value	HR (95% CI)	
**Age, years**		0.398		
≤50	89.1			
>50	85.5			
**Sex**		0.185		
Male	86.8			
Female	91.7			
**T classification**		0.068		
T1–3	89.4			
T4	81.2			
**N classification**		0.003		0.005
N0–1	90.5		1	
N2–3	81.6		2.246 (1.276–3.955)	
**Serum EBV DNA, copies/mL**		0.003		0.004
≤4000	91.7		1	
>4000	81.9		2.321 (1.317–4.089)	
**Leukocytes, k/cc**		0.012		
≤10	89.0			
>10	73.4			
**Hemoglobin, g/L**		0.978		
≤120	88.5			
>120	87.9			
**Platelets, k/cc**		0.016		0.007
≤300	89.5		1	
>300	76.2		2.531 (1.288–4.977)	
**Serum albumin, g/L**		0.009		0.008
≤46	84.2		2.290 (1.244–4.214)	
>46	92.1		1	
**Serum alkaline phosphatase, U/L**		0.681		
≤110	88.2			
>110	85.0			
**Serum lactate dehydrogenase, U/L**				
≤245	87.6	0.222		
>245	100			

Abbreviations: CCRT, concurrent chemoradiotherapy; 95% CI, 95% confidence interval; HR, hazard ratio.

**Table 3:** Stratification survival analysis of the CCRT group versus the NACT + CCRT group (*n* = 881).

Subgroups	Distant metastasis-free survival		Progression-free survival	
	Rate at 3 years (CCRT group vs. NACT + CCRT group)	*P-*value	Rate at 3 years (CCRT group vs. NACT + CCRT group)	*P-*value
**N classification**				
N0–1 (*n* = 588)	90.5 vs. 93.1	0.356	85.5 vs. 86.2	0.956
N2–3 (*n* = 293)	81.4 vs. 82.6	0.630	73.1 vs. 75.9	0.237
**Serum EBV DNA level, copies/mL**				
≤4000 (*n* = 445)	91.7 vs. 94.5	0.281	85.8 vs. 87.0	0.802
>4000 (*n* = 436)	81.6 vs. 85.7	0.289	75.6 vs. 79.3	0.228
**Platelets, k/cc**				
≤300 (*n* = 745)	89.5 vs. 90.2	0.814	83.1 vs. 82.8	0.979
>300 (*n* = 136)	76.2 vs. 84.7	0.264	73.9 vs. 80.1	0.363
**Serum albumin, g/L**				
≤46 (*n* = 366)	84.2 vs. 89.4	0.070	77.3 vs. 82.1	0.158
>46 (*n* = 515)	92.1 vs. 88.8	0.197	87.2 vs. 82.8	0.322
**Prognostic score model**				
Low risk (score 0–1, *n* = 427)	93.1 vs. 91.6	0.411	88.5 vs. 85.0	0.199
High risk (score 2–4, *n* = 454)	80.3 vs. 87.6	0.025	72.5 vs. 80.7	0.013

Abbreviations: CCRT, concurrent chemoradiotherapy; NACT, neoadjuvant chemotherapy.

**Table 4:** Multivariate analysis of distant metastasis and tumor progression in high-risk patients with locally advanced NPC (*n* = 454).

Variable	Distant metastasis-free survival		Progression-free survival	
	*P*-value	HR (95% CI)	*P*-value	HR (95% CI)
**N classification** (N2–3 vs. N0–1)	0.001	2.490 (1.443–4.298)	0.018	1.637 (1.087–2.465)
**Chemotherapy** (CCRT vs. NACT + CCRT)	0.001	2.338 (1.408–3.881)	0.011	1.657 (1.123–2.446)
**Serum lactate dehydrogenase** (>245 vs. ≤ 245 U/L)	0.018	2.187 (1.141–4.191)	0.077	1.702 (0.943–3.071)
**Serum EBV DNA level** (>4000 vs. ≤4000 copies/ml)	0.009	2.376 (1.239–4.557)		
**Platelets** (>300 vs. ≤ 300 k/cc)	0.006	2.118 (1.245–3.604)		
**Serum albumin** (≤46 vs. > 46 g/L)	0.079	1.781 (0.935–3.392)		

Abbreviations: CCRT, concurrent chemoradiotherapy; NACT, neoadjuvant chemotherapy.
